# Trajectory Discrimination and Peripersonal Space Perception in Newborns

**DOI:** 10.1111/infa.12207

**Published:** 2017-08-23

**Authors:** Giulia Orioli, Maria Laura Filippetti, Walter Gerbino, Danica Dragovic, Teresa Farroni

**Affiliations:** ^1^ Department of Developmental Psychology and Socialization (DPSS) University of Padua; ^2^ Research Department of Clinical, Educational and Health Psychology University College London; ^3^ Department of Life Sciences – Psychology Unit “Gaetano Kanizsa” University of Trieste; ^4^ Department of Paediatric Unit Hospital “S. Polo” ‐ Monfalcone

## Abstract

The ability to discriminate the trajectories of moving objects is highly adaptive and fundamental for physical and social interactions. Therefore, we could reasonably expect sensitivity to different trajectories already at birth, as a precursor of later communicative and defensive abilities. To investigate this possibility, we measured newborns' looking behavior to evaluate their ability to discriminate between visual stimuli depicting motion along different trajectories happening within the space surrounding their body. Differently from previous studies, we did not take into account defensive reactions, which may not be elicited by impending collision as newborns might not categorize approaching stimuli as possible dangers. In two experiments, we showed that newborns display a spontaneous visual preference for trajectories directed toward their body. We found this visual preference when visual stimuli depicted motion in opposite directions (approaching vs. receding) as well as when they both moved toward the peripersonal space and differed only in their specific target (i.e., the body vs. the space around it). These findings suggest that at birth human infants seem to be already equipped with visual mechanisms predisposing them to perceive their presence in the environment and to adaptively focus their attention on the peripersonal space and their bodily self.

Being able to discriminate the trajectories of moving objects is highly adaptive, as well as crucial for the implementation of successful physical and social interactions. This ability becomes essential when objects move toward the observer: An approaching object is predictive of communication and/or physical contact, and it might represent a threat or danger (Kandula, Hofman, & Dijkerman, 2015; Vagnoni, Lourenco, & Longo, 2012; Yonas et al., 1977). Importantly, the discrimination of approaching objects becomes fundamental when they enter the observers' peripersonal space (PPS), that is, the portion of space immediately surrounding the body, which mediates all physical and social interactions between the body and the adjacent environment (Canzoneri, Magosso, & Serino, 2012; de Vignemont & Iannetti, 2015).

Several studies in human adults highlighted the importance of perceiving and discriminating approaching trajectories (Abrams & Christ, 2005, 2006; Cappe, Thut, Romei, & Murray, 2009; Franconeri & Simons, 2003, 2005; Ghazanfar, Neuhoff, & Logothetis, 2002; Neuhoff, 1998, 2001; Rosenblum, Carello, & Pastore, 1987; Rosenblum, Wuestefeld, & Saldaña, 1993; Rossini, 2014; Seifritz et al., 2002). These studies used either visual or auditory signals (or a combination of the same) and showed that looming stimuli—which specify impending collision (Howard & Rogers, 2008)—receive a privileged processing. They also suggested that such facilitation should be interpreted according to the potential evolutionary benefit that prioritizing the processing of auditory or visual looming signals could provide, helping to create and maintain a margin of safety around the body (Ghazanfar et al., 2002). For example, research using auditory looming showed that adults systematically underestimate the arrival time of a sound characterized by rising intensity (Rosenblum et al., 1987), that they perceive rising (i.e., approaching) sounds as closer to themselves compared to falling (i.e., receding) sounds (Rosenblum et al., 1993), and that they overestimate the change in intensity level of rising vs. falling sounds (Neuhoff, 1998, 2001). Research that focused on the visual modality also provided evidence of the evolutionary importance of looming stimuli, demonstrating that looming motion can capture attention to a higher extent than receding motion (Abrams & Christ, 2005, 2006; Franconeri & Simons, 2003, 2005) and that the processing of looming stimuli is prioritized by the visual system (Rossini, 2014).

Sensitivity to impending collision—signaled by visual looming stimuli—has been investigated also in infancy. Previous infant studies used different methods and focused on different aspects of infants' responses to impending collision, but shared the assumption that impending collision signals an upcoming threat, whose perception should trigger a defensive reaction (Kayed & van der Meer, 2000, 2007). The presence and timing strategies of defensive eye blinks have been investigated, as blinking has been considered “the best indicator of awareness of the collision course of an object in infancy” (Kayed & van der Meer, 2000, p. 254; Kayed & van der Meer, 2007; Yonas, 1981). Yonas et al. (1977) traced the development of avoidant responses to impending collision information in 1‐ to 9‐month‐old infants. Using eye blinking as a measure of sensitivity to looming stimuli, they suggested that sensitivity to impending collision undergoes an extended developmental course and emerges from 4 to 6 months of age, when infants' blinking response to approaching visual stimuli becomes consistent (Yonas et al., 1977). Similarly, Schmuckler, Collimore, and Dannemiller (2007) examined which aspects of visual information effectively trigger defensive responses to impending collision in infants. They showed that 4‐ and 5‐month‐old infants blink significantly more in response to approaching compared to withdrawing movements and that, among approaching trials, movements approaching from the center vs. from the side tend to trigger more eye blinks, especially if anticipating a collision. Furthermore, they demonstrated that among trials showing an approach from the side, eye blinks seem to be more frequent when the object crossed in front of the infant vs. when it did not. More recently, a few studies investigated the strategies used by infants to time a defensive eye blink and identified the emergence of a sophisticated timing strategy, based on the computation of the expected time‐to‐collision of the approaching object, around 6 months of age (Kayed & van der Meer, 2000, 2007). Developmental studies have also investigated the neural correlates of visual looming processing, with a particular focus on how infants' brain processes timing information on the approach of a potentially dangerous visual stimulus (van der Meer, Svantesson, & van der Weel, 2012; van der Weel & van der Meer, 2009). These studies showed the presence of theta band event‐related oscillations in the left visual cortex in response to looming stimuli and the existence, at 10 and 11 months, of a well‐established neural network for processing impending collision (van der Weel & van der Meer, 2009). Additionally, they found, in 12‐ and 13‐month‐old infants, shorter visual evoked potential (VEP) peaks that happened closer to the time‐to‐contact of the looming stimulus. They suggested that this is indicative of a developmental shift in the processing of looming stimuli, further supported by the propagation of the peak VEP activation toward higher processing areas in older infants (van der Meer et al., 2012).

Defensive reactions to looming stimuli—considered as signifiers of an imminent danger—have been also used to assess the sensitivity to stimuli approaching along a colliding pathway in newborn infants (Ball & Tronick, 1971; Bower, Broughton, & Moore, 1971; Náñez, 1988; Yonas et al., 1977). Bower et al. (1971) identified the presence of a sporadic and posture‐dependent adaptive response, specifically modulated by the visual components of the looming stimuli. In their work, newborns performed a specific set of behaviors in response to these stimuli, comprising eye widening, withdrawal of the head and movement of the hands between the colliding object and the face (Bower et al., 1971). Similarly, Ball and Tronick (1971) recorded backwards head movements in response to looming stimuli directly targeting the newborns' body, and head turning following looming stimuli approaching the newborns but missing them laterally. However, Yonas et al. (1977) later questioned these findings. They studied the development of sensitivity to impending collision in the first year of life, by measuring both postural responses and eye blinks and demonstrated that postural changes should not be considered as defensive behaviors, but rather as “an aspect of tracking” (Yonas et al., 1977, p. 98) of the moving object. Furthermore, they showed that in the first months of life, infants do not show defensive behaviors, which develop gradually during the first year of life. In light of the absence of avoidant behaviors in 1‐ and 2‐month‐old infants, Yonas and colleagues concluded that these young infants do not show sensitivity to visual information specifying impending collision.

Altogether, the absence of avoidant responses to impending collision trajectories found in the aforementioned studies led to the conclusion that sensitivity to approaching motion itself is not present at birth and develops gradually during the first year of life. However, the analysis of defensive behaviors originates from the assumption that approaching trajectories necessarily anticipate an upcoming danger, and therefore from the idea that infants attribute a negative, threatening value to these trajectories. Crucially, the information conveyed by an approaching stimulus does not necessarily signal an upcoming danger, but could rather indicate an interesting stimulus with which it would be possible to interact (de Vignemont & Iannetti, 2015; Kandula et al., 2015; Van der Stoep, Nijboera, Van der Stigchel, & Spence, 2015). Therefore, we suggest that the presence vs. the absence of defensive reactions may not have been the most informative variable to consider when investigating sensitivity to approaching trajectories information in newborn infants, as the discrimination of the different trajectories could be present irrespective of defensive reactions. Indeed, research on optical flow investigated newborns' sensitivity to visual motion information without necessarily attributing a negative valence to it (Barbu‐Roth et al., 2009, 2014; Jouen, 1990; Jouen, Lepecq, Gapenne, & Bertenthal, 2000). These studies demonstrated that newborns as young as 3 days are sensitive to peripheral optical flow. In fact, when presented with bilateral backwards optical flow (which in adults is associated with forward locomotion in a static environment), they perform backwards compensatory head movements, whose speed is sensitive to the speed of the displayed optical flow (Jouen et al., 2000). Another set of studies measured how newborns' stepping reflex is modulated by optical flow (Barbu‐Roth et al., 2009, 2014). They suggested the existence at birth of a first form of coupling between air‐stepping and visual motion, demonstrated by the enhanced stepping responses to translating vs. rotating or static visual stimuli (Barbu‐Roth et al., 2009). Furthermore, they showed that newborns' stepping reflex was modulated by the direction of the optical flow, with more knee joint activity in response to a pattern that moved toward rather than away from them (Barbu‐Roth et al., 2014). Altogether, these findings suggest that soon after birth newborns exhibit simple reactions to optical flow, showing a rudimentary processing of visual motion information that specifies self‐motion. Based on this evidence, we hypothesized that focusing on the lack of a defensive response could have masked very young infants' ability to discriminate among relevant moving trajectories. In fact, as there is no evidence of humans' ability to recognize stimuli as dangerous soon after birth (Farroni, Menon, Rigato, & Johnson, 2007; Johnson, Senju, & Tomalski, 2015), we reasoned that the absence of the blinking reflex to impending collision in newborns might not be due to their lack of sensitivity to motion trajectory. Rather, soon after birth infants might not categorize approaching stimuli as a possible danger in the first place, and therefore, they might not show avoidant behaviors in response to them.

The purpose of our study was to track the developmental origins of the facilitated processing of approaching stimuli, which is apparent in adults (Abrams & Christ, 2005, 2006; Cappe et al., 2009; Franconeri & Simons, 2003, 2005; Neuhoff, 1998, 2001; Rosenblum et al., 1987, 1993; Rossini, 2014; Seifritz et al., 2002). In fact, considering the important adaptive features of stimuli approaching one's own body along a colliding pathway—for both defensive and communicative purposes—it would be reasonable to expect that soon after birth infants could be already sensitive to motion along this trajectory. In light of this expectation, we wanted to investigate whether soon after birth, when the experience of the world is minimal, newborns could discriminate between different motion trajectories taking place in the space surrounding their bodies and whether they might devote more attention to those conveying adaptively relevant information. In fact, it could be hypothesized that very young infants may present a perceptual bias toward motion approaching the body vs. directed elsewhere. Such bias could predispose newborns to focus their attention on the space immediately surrounding their bodies and on the events taking place within it, thus driving their visual attention toward relevant information available in the environment (Lakusta & DiFabrizio, 2017). Relatedly, the existence of such bias might also be important for subsequent development as it might play a role in infants' learning processes (Lakusta & DiFabrizio, 2017).

In order to address this tempting hypothesis, we measured newborns' looking behavior to stimuli moving along different trajectories within the space surrounding their bodies. Through the use of a preferential looking paradigm, we aimed to investigate the existence of a spontaneous visual preference for adaptively relevant trajectories, like those approaching the newborns along a colliding pathway. We designed a preferential looking experiment comparing a visual stimulus depicting an approaching and colliding trajectory (AC) with a visual stimulus depicting a receding trajectory (R). We expected that the newborns would show a significant visual preference for the AC trajectory, which is adaptively more relevant as it is directed toward their body. We also wanted to generalize our findings to other trajectories and to test whether the hypothesized preference for the one approaching the body could be specifically related to the impending collision depicted by it, or more generally to approach or expansion in optical size. Therefore, we included a further sequential looking experiment comparing, across two trials, two visual stimuli both depicting approaching trajectories, one colliding (AC) and one non‐colliding (ANC). Here, both stimuli approached the newborns' PPS and both increased in optical size, but only one trajectory specifically targeted their body. Again, we expected the newborns to look longer at the trajectory where the moving object directly approached their body, that is, AC. Importantly, this experiment also gave us the opportunity of investigating whether newborns could discriminate between two trajectories both moving toward the same portion of space (i.e., the PPS) and differing only in their specific target (i.e., the body vs. the space around it).

We hypothesized that the evidence derived from the looking times in the two experiments would provide us with information about newborns' implicit and rudimentary ability to differentiate the space surrounding their own body. We expected to replicate previous findings showing the absence of consistent blinking in response to impending collision at birth. Crucially, we expected to find a visual preference for the approaching and colliding trajectory, which could be more interesting and adaptively more relevant than trajectories directed somewhere else, as it would lead to an interaction—either positive or negative—between the newborn and the moving stimulus.

## Methods

### Participants

The study was conducted at the Paediatric Unit of the Hospital of Monfalcone (GO ‐ Italy), where all the newborns were born. Twenty newborns (seven female) aged between 14 and 95 h at time of test took part in the study. Ten additional newborns participated, but were excluded due to fussiness (*n* = 4), sleepiness (*n* = 2), or a strong side bias (i.e., they oriented more than 80% of the time to the same side, *n* = 4). All the newborns met the screening criteria for normal delivery, birth weight >2,500 g, gestational age >37 weeks and Apgar score ≥8 at 5 min of life. No abnormalities were present at birth. The 20 newborns included in the final sample had a mean age of 46.53 h (*SD* = 22.16), a mean birth weight of 3,358.5 g (*SD* = 443.43), and a mean gestational age of 40.50 weeks (*SD* = 0.92).

Testing took place when babies were awake and alert, usually during the hour preceding feeding time. Parents were informed about the procedure and Informed Consent to their child's participation was obtained from them. The local Ethical Committee of Psychology Research (University of Padua) approved the study protocol.

### Apparatus

The newborns sat on the experimenter's lap and watched the stimuli presented on a monitor (24″) in front of them. The distance between the newborns' face and the monitor was about 30 cm, distance at which visual acuity at birth is better (Fantz, Ordy, & Udelf, 1962; Slater, 2002). Black cardboard and black curtains covered the area around the monitor to prevent external stimuli from engaging the newborns' attention. The newborns' eye level was aligned to the center of the screen. A video camera located on top of the screen recorded the newborns' eyes allowing following offline coding of their eye movements. Stimuli were presented using E‐Prime 2.0.10.

### Procedure and stimuli

Each newborn took part in both experiments. The order of presentation of the experiments and of the trials within each experiment was counterbalanced across participants. The experiment began as soon as the newborn was seated and was attending to the center of the screen.

**Figure 1:**
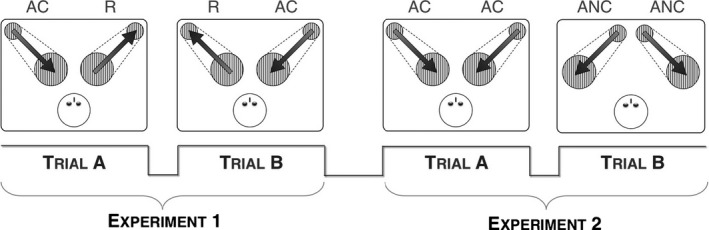
Experimental procedure. Each newborn participated in two experiments, one immediately following the other. The two experiments and the two trials within each experiment were presented in counterbalanced order across participants. In Experiment A, in each trial, we compared parallel stimuli depicting an approaching and colliding (AC) and a receding (R) trajectory (eight presentations); the positions of the two stimuli were counterbalanced across trials. In Experiment B, in one trial (A), we presented two simultaneous stimuli depicting an AC trajectory (eight presentations), whereas in another trial (B), we presented two simultaneous stimuli depicting an approaching but non‐colliding (ANC) trajectory (eight presentations). In Experiment B, the Looking Times were compared across trials (sequential looking time).

In Experiment A, the newborns were presented with a visual stimulus depicting an approaching and colliding trajectory (AC) compared to visual stimulus depicting a receding trajectory (R). In Experiment B, instead, the visual stimuli presented to the newborns depicted two different approaching trajectories, moving either along a colliding (AC) or non‐colliding pathway (ANC). In both experiments, the newborns were presented with two simultaneous visual stimuli, one on the left and one on the right of the screen, in order to ensure that their attention was engaged. Experiment A used a parallel preferential looking paradigm (Fantz, 1956, 1958), thus the newborns were presented with two different stimuli on the two sides of the screen, with counterbalanced positions across trials. Experiment B, instead, used a sequential looking paradigm, in which the visually presented trajectories were always symmetrical with respect to the body midline, in order to obtain an absolute measure of visual preference, controlling for artifacts that could affect the relative attractiveness of adjacent stimuli themselves (e.g., size or speed). Hence, the newborns were presented with different stimuli in the two trials, but with the same stimulus on both sides of the screen in each trial. Here, the looking times to the different stimuli were compared between trials (Figure 1 and Movies S1–S4 in the Supporting Information online).

The videos had been previously recorded in an ecological context and showed the movement of a black‐and‐white‐striped ball along each of the three trajectories. The recorded ball had a diameter of 8 cm and a pattern of vertical black and white stripes, 1 cm wide, and moved at constant speed in 3D space. The recording structure was chosen accordingly to monitor size (24″). The camera was placed where the newborns' head would be positioned during the experiment, that is, pointing toward the center of the recording structure (both horizontally and vertically) at 35 cm from it.

During the experiment, the monitor (24″) showed a gray background containing two peripheral black areas, where the stimuli appeared. Both were 24.4 cm wide and 20.4 cm high; they were both 0.85 cm apart from the nearest edge of the screen and 1.6 cm apart from one another; they were both 6 cm apart from the top and the bottom of the screen. At the beginning of AC and ANC stimuli and at the end of the R one the ball subtended a visual angle of 23.54° × 23.54°; on average, the width of the stripes subtended a visual angle of 2.94°. The ball was 3.2 cm apart from the nearest edge of the frame and 14 cm apart from the farthest and was 6.6 cm apart from the top and the bottom of the frame. At the end of the AC stimulus and at the beginning of the R one, the ball subtended a visual angle of 37.70° × 37.70°; on average, the width of the stripes subtended a visual angle of 4.71°. The ball was 5.3 cm apart from both edges of the frame and was 3.3 cm apart from the top and the bottom of the frame. At the end of the ANC stimulus, the ball subtended a visual angle of 34.70° × 34.70°; on average, the width of the stripes subtended a visual angle of 4.33°. The ball was 3.2 cm apart from the nearest edge of the frame and 11.2 cm apart from the farthest and was 5.2 cm apart from the top and the bottom of the frame. The luminance of the display was 0.5 cd/m^2^ for the black frames and 54 cd/m^2^ for the gray background; it was instead 78 cd/m^2^ for the white stripes of the moving ball and 108 cd/m^2^ for the lightest part of it (highlight). We provided the infants with high contrast stimuli in order to enhance their attention toward them; in fact, Michelson contrast between the black frames and the gray background was −0.982, between the white stripes of the ball and the black frames it was 0.987, and between the highlight of the ball and the black frames it was 0.991. The black curtain surrounding the monitor had a luminance of 0.2 cd/m^2^ and the room was poorly lit in order to ensure that the newborns' attention was focused toward the screen (average walls luminance was 30 cd/m^2^; average ceiling luminance was 15 cd/m^2^). All measures were taken from the infant's position during the experiments, and the ambient lighting while measuring was the same as the average lighting of the room while testing was conducted. The speed of our stimuli resulted from the combination of the length of the path that the recorded ball had to travel (35.51 cm for AC and R; 30 cm for ANC) and a display time long enough to ensure that the newborns' attention could be engaged. The resulting speed of the ball in the AC and R stimuli was 10.6 cm/sec, whereas in the ANC stimulus, it was 9 cm/sec. We were not concerned about the effect that the speed of the moving stimulus could have on the discrimination of the different trajectories. In fact, previous studies did not use stimuli moving with a consistent speed and their results indicated that even wide variations in the looming speed (i.e., from 6 to 48 cm/sec) did not have any impact on infants' reactions (Náñez, 1988). Each stimulus moved for 3,333 msec, preceded and followed by 10 frames (333 msec) where the ball stood still (during the last one contrast was reduced, favoring a fading effect), for an overall stimulus duration of 4,000 msec. Each stimulus was presented eight times, with a 1‐sec interval between two subsequent presentations and 4,000 msec of blank screen before the first presentation, for an overall trial duration of 44 sec.

### Looking behavior analysis

The video recordings of the newborns' eye movements were analyzed offline by two independent observers. The second coder was unaware of the hypotheses, and both were blind to experiments and trials order. They coded how long each newborn looked at each side of the screen during both experiments. In this way, we obtained a measure of the time that the newborns spent looking at each stimulus. For each newborn, we calculated the proportions of Looking Time [P(LT)] directed to each stimulus dividing the LT to each stimulus by the total exposure time of that stimulus (Table 1). Two inter‐rater reliability analyses were performed. Pearson's *r* correlation was performed on the total sample and revealed a score of *r *=* *0.90; interclass correlation coefficient was measured for 20% of the sample (*n* = 4) and showed an agreement between coders = 0.94.

**Table 1:** Looking Time Results

Experiment	Trajectory	Average LT (msec)	Exp (msec)	P(LT)	*SE*	*t*	df	*p*	*d*_z_
A	AC	17,332	64,000	.2708	.0127	3.602	19	.006	0.68
	R	12,770	64,000	.1995	.0157				
B	AC	18,204	32,000	.5689	.0282	5.139	19	<.001	1.15
	ANC	14,853	32,000	.4642	.0278				

The table shows the average raw looking times (LT) to each stimulus in the two experiments, the total exposure times (Exp), the proportions of looking time [P(LT)], calculated dividing the LT to each stimulus by the total exposure time of that stimulus, and their Standard Error (SE).

## Results

The data from each experiment were analyzed using mixed ANOVAs. For Experiment A, the ANOVA explored the effect of the Stimulus (within participants) and of the Experiment order (between participants) on the P(LT). For Experiment B, instead, it explored the effect of the Stimulus (within participants) and of the order of presentation of the Experiments and of the Trials within Experiment B (between participants) on the P(LT). By investigating the effects of the order of presentation of the Experiments and, in Experiment B, also of the Trials within the experiment, we wanted to exclude the possibility that the looking time in the experiment presented as second could be influenced by the stimuli attended in the experiment presented as first. In particular, in our design, the visual stimulus depicting an approaching and colliding trajectory was presented in both experiments, whereas the stimuli depicting the receding and the approaching but non‐colliding trajectories were presented only in one experiment each. Therefore, we wanted to verify that the looking time toward the approaching and colliding stimulus in the experiment presented as second was not influenced by the fact that the newborns had previously been exposed to the same visual stimulus.

**Figure 2:**
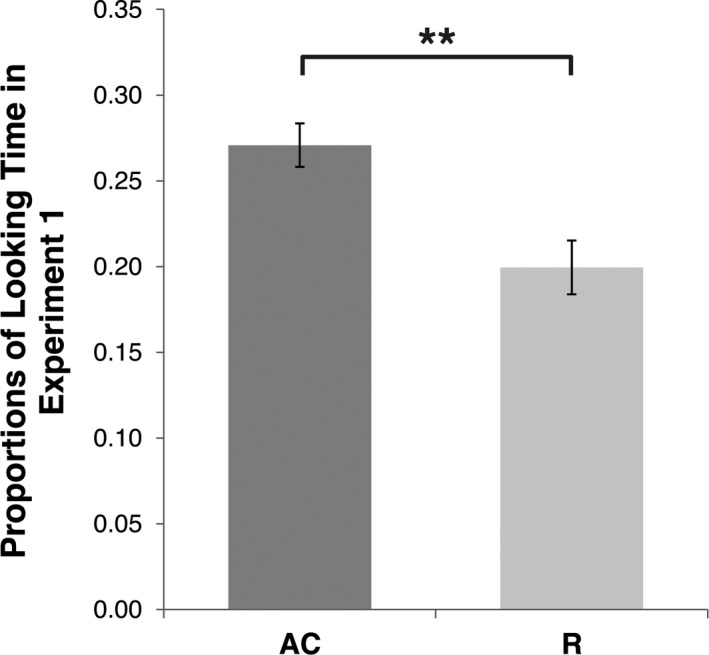
Looking Time results in Experiment A. Mean and *SE* of the proportion of Looking Time [P(LT)] directed to approaching and colliding (AC) and receding (R) visual stimuli during Experiment A. ** p < 0.01

In Experiment A, the ANOVA revealed a significant main effect of the Stimulus [*F*(1, 18) = 8.892, *p *=* *.008, *η* ^2^ **= .330] and no significant effects of the Experiment order [*F*(1, 18) = .667, *p *=* *.425, *η* ^2^ **= .036] nor of the Interaction between the two factors [*F*(1, 18) = .025, *p *=* *.876, *η* ^2^ **< .001]. Then, in light of these results, we compared, using a paired samples *t*‐test, the P(LT) toward the two stimuli, irrespective of the order of presentation of the two experiments. The distribution of the differences in P(LT) to the two stimuli did not deviate significantly from normality [Kolmogorov–Smirnov test: *D*(20) = .183, *p *=* *.077]. The *t*‐test showed that the newborns looked significantly longer to the AC stimulus compared to the R stimulus [*t*(19) = 3.062, *p *=* *.006; *d*_z_ = 0.68] (Figure 2), showing a visual preference for a stimulus depicting an approaching and colliding trajectory vs. one depicting a receding trajectory.

**Figure 3:**
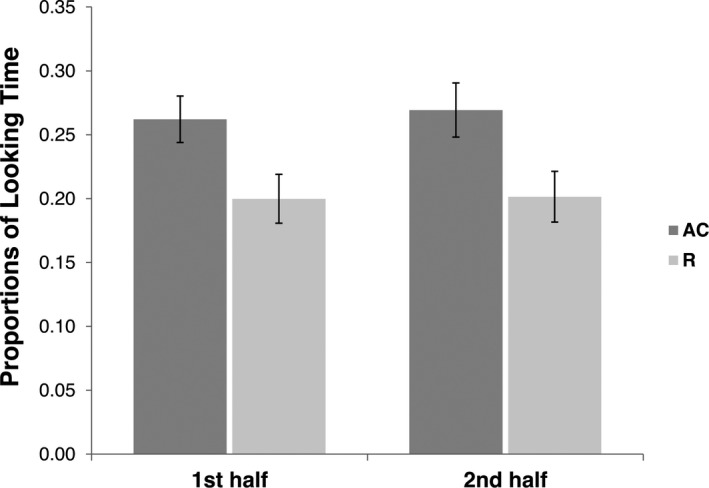
Looking Time results. Mean and *SE* of the proportions of Looking Time [P(LT)] to approaching and colliding (AC) and receding (R) stimuli during the first and the second halves of each presentation (Experiment A).

We ran an additional ANOVA to investigate whether this visual preference was affected by a preference for the bigger stimulus. In fact, in the first half of the presentation, the R stimulus had a larger optical size than the AC stimulus, whereas the opposite was true in the second half of the presentation. To investigate the possible effect of optical size on the newborns' looking behavior, we compared the P(LT) to AC vs. R stimuli in the first vs. second half of each of the eight presentations included in each trial (i.e., LT from 0 to 2,000 msec vs. LT from 2,000 to 4,000 msec). The results showed a main effect of the Stimulus [*F*(1, 38)* *= 4.744, *p *=* *.036; *η* ^2^ **= .108], but no effect of the Presentation half [*F*(1, 38)* *= .246, *p *=* *.623; *η* ^2^ **= .006] nor of the Interaction [*F*(1, 38)* *= .032, *p *=* *.860; *η* ^2^ **= .001]. This showed that the AC stimulus was attended for a longer proportion of time compared to the R one in both the first and the second halves of the presentations and that the amount of looking time directed to each stimulus in first vs. second half of the presentations was not significantly different (Figure 3). Hence, the visual preference for AC shown in this experiment did not depend on the dimension of the stimuli, but rather on the trajectory that they depicted, as demonstrated by the fact that the newborns looked longer to the AC stimulus since the very beginning of its presentation.

**Figure 4:**
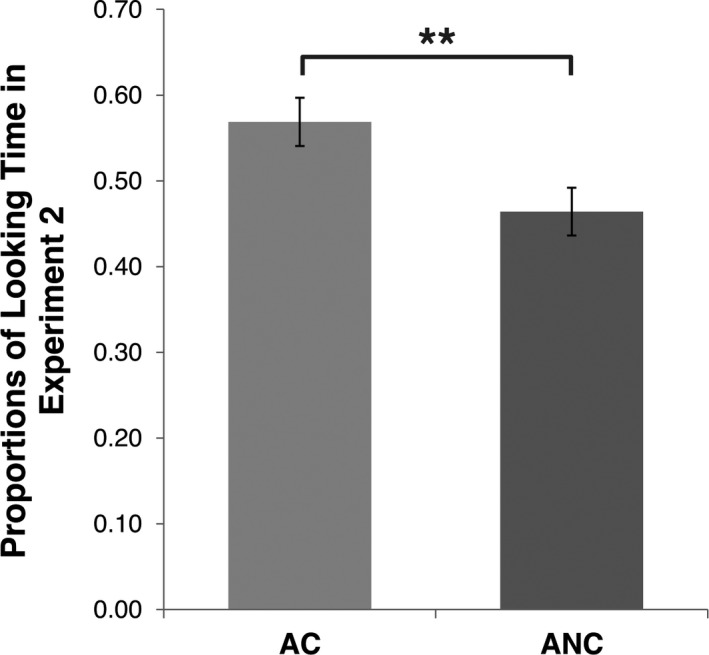
Looking Time results in Experiment B. Mean and *SE* of the proportion of Looking Time [P(LT)] directed to approaching and colliding (AC) and approaching but non‐colliding (ANC) visual stimuli during Experiment B. ** p < 0.01

Experiment B was introduced in order to generalize the investigation to different trajectories, as well as to rule out the possibility that the preference for approaching stimuli in Experiment A was determined only by a preference for expanding vs. contracting stimuli, which would not necessarily take into account either motion in depth or impending collision information. The ANOVA revealed a significant main effect of the Stimulus [*F*(1, 16) = 22.731, *p *<* *.001, *η* ^2^ **= .582] and no significant effect of Experiment order [*F*(1, 16) = .784, *p *=* *.389, *η* ^2^ **= .037] and Trial order [*F*(1, 16) = .001, *p *=* *.980, *η* ^2^ **< .001], nor of any of the Interactions [Stimulus * Experiment order: *F*(1, 16) = .122, *p *=* *.732, *η* ^2^ **= .003; Stimulus * Trial order: *F*(1, 16) = .230, *p *=* *.638, *η* ^2^ **= .006; Experiment order * Trial order: *F*(1, 16) = 4.240, *p *=* *.056, *η* ^2^ **< .001; Stimulus * Experiment order * Trial order: *F*(1, 16) = .004, *p *=* *.952, *η* ^2^ **= .330]. In light of these results, we ran a paired samples *t*‐test in order to compare the P(LT) toward the two stimuli, irrespective of the order of presentation of the two experiments and of the two trials within Experiment B. The distribution of the differences in P(LT) to the two stimuli did not deviate significantly from normality [Kolmogorov–Smirnov test: *D*(20) = .149; *p *=* *.200]. The *t*‐test showed that, in line with the results of Experiment A, the newborns showed a visual preference for the AC stimulus compared to the ANC stimulus [*t*(19) = 5.139; *p *<* *.001; *d*_z_ = 1.15] (Figure 4). Thus, the results of Experiment B demonstrated: (1) newborns' ability to discriminate the specific target of an approaching trajectory, and (2) a visual preference for the stimuli on a collision course with their own body.

Prior work investigating newborn infants' perception of object trajectories in relation to themselves has focused on their defensive behaviors (Ball & Tronick, 1971; Bower et al., 1971; Kayed & van der Meer, 2000, 2007; Náñez, 1988; Schmuckler et al., 2007; Yonas, 1981; Yonas et al., 1977). Therefore, we also coded the number of eye blinks shown by our sample of newborns in trials presenting at least one stimulus depicting a colliding trajectory (Experiment A, both trials; Experiment B, AC‐AC trial only). We used the definition of defensive eye blink employed in previous studies (i.e., whether the eyes are closed within 1 sec either before or after the collision; Schmuckler et al., 2007; Yonas et al., 1977). We recorded a total of 18 eye blinks of 480 colliding stimuli displayed to the whole sample of newborns (4%); among those only 6 happened when the newborn was looking at the AC stimulus before closing her/his eyes.

## Discussion

In these two experiments, we have shown that newborn infants can discriminate between the trajectories of moving stimuli, as demonstrated by their visual preference for those directed toward their own bodies. This visual preference for the trajectory targeting their own body was shown not only when the displayed trajectories moved in opposite directions (approaching vs. receding), but also when they both approached their PPS. By measuring preferential looking behavior, this is the first study to show that soon after birth infants are able to make quite sophisticated perceptual discriminations of visual trajectories approaching their body vs. trajectories either receding from them or approaching the space around their body (PPS), but not on a direct collision course.

Previous research on the sensitivity to impending collision in newborns concluded that very young infants are not sensitive to the colliding course of a moving object, based on the absence of adaptive responses or defensive behaviors (i.e., lack of eye blinks) (Yonas et al., 1977). Here, we hypothesized that focusing on defensive responses, such as the blinking reflex, could have masked infants' ability to detect and discriminate among moving trajectories. In fact, the blinking reflex may not be elicited by impending collision in newborns because they might not categorize looming stimuli as a possible danger, rather than because they are not sensitive to their motion trajectory. Conversely, they might show a perceptual bias toward trajectories approaching the body, which could signal not only an impending danger, but also an approaching interesting object (de Vignemont & Iannetti, 2015; Kandula et al., 2015; Van der Stoep et al., 2015), and that infants must be able to process in order to functionally respond to the surrounding environment (Lakusta & DiFabrizio, 2017). Here, we used a classic behavioral measure capable of detecting attentional responses in pre‐verbal infants with the aim of testing the existence of this perceptual bias soon after birth.

We implemented two looking behavior experiments in order to evaluate newborns' ability to discriminate between different trajectories, by hypothesizing the existence of a spontaneous preference for approaching and colliding ones. In Experiment A, we compared a trajectory approaching along a collision course with a receding trajectory, whereas in Experiment B, we investigated newborns' discrimination of two trajectories both moving toward the space around the body and only differing in their specific target (i.e., the body vs. the space surrounding it). In both experiments, we found a visual preference for the approaching and colliding stimulus. This result is consistent with the hypothesis that newborns can discriminate the actual trajectory of moving stimuli with respect to the body and show a visual preference for those trajectories targeting their body, that is, approaching and colliding trajectories. Crucially, the newborns showed this visual preference for the trajectory targeting their own body both when the stimuli depicted motion in opposite directions and when they both approached their PPS.

In Experiment A, we compared approaching and colliding vs. receding stimuli and showed that the newborns looked longer to the approaching and colliding ones. Based on the findings of Experiment A alone, it could be argued that the visual preference that we found could be attributed to a general preference for approach and not specifically to the presence of impending collision information. At the same time, it could be referred to a preference for the increasing optical size of the approaching stimulus, rather than to its trajectory. To address these issues, in Experiment B, we compared two different approaching trajectories: This time, both stimuli approached the newborns and increased in optical size, but only one specified an impending collision. The visual preference for approaching and colliding rather than approaching but non‐colliding stimuli in Experiment B confirmed the visual preference for approaching and colliding stimuli found in Experiment A. This suggests that newborns' preference for stimuli depicting trajectories directed toward their body is due to a real preference for impending collision information, rather than to a preference for increasing optical size or for approach in general. Importantly, it also shows newborns' sophisticated ability to visually discriminate between two stimuli depicting trajectories that are both moving toward the space immediately surrounding their body, that is, their PPS. Additionally, our results ruled out the existence of any effects of the order of presentation of the experiments and, for Experiment B, of the trials within the experiment. Most importantly, they excluded the possibility that the order of presentation interacted with the looking times to the visual stimulus depicting an approaching and colliding trajectory. This means that in neither of the two experiments, the looking time toward the approaching and colliding visual stimulus was influenced by a potential familiarity effect with the same stimulus when such experiment was presented as second.

Together, these results support our hypothesis that already at birth humans are able to discriminate between different motion trajectories. Furthermore, the specific direction of the preference in both experiments indicates that newborns have a particular interest in stimuli directly approaching their own body. This preferential looking seems to suggest the existence, at birth, of a predisposition to perceive one's own presence in the environment and to adaptively focus attention on one's own body and on the space around it. Such early predisposition could influence infants' subsequent development, having a role in their learning processes by directing their attention to the most relevant information present in the environment (Lakusta & DiFabrizio, 2017).

We speculate that the preference for approaching and colliding trajectories could be ascribed to the higher adaptive value of a stimulus that, moving along a collision course, could come into direct contact with the newborns. The stimulus could either have a positive (interaction) or negative (danger) value, but, in both instances, it appears to be worth looking at it. Preferential looking paradigms cannot provide any information about the positive or negative valence of the shown stimuli, or the reason why one stimulus is visually preferred over the other (Banks & Ginsburg, 1985). As a consequence, based on the present data, we are unable to draw a definite conclusion on whether the longer looking time directed to the approaching and colliding stimulus was due to interest or threat. Additional studies using physiological measurements are needed in order to shed further light on the valence of a stimulus approaching along a colliding trajectory in infancy. At the same time, the positive or negative salience of a stimulus moving into the space surrounding the body could be directly linked to the two alternative functions that characterize this space itself (de Vignemont & Iannetti, 2015). de Vignemont and Iannetti (2015) recently differentiated between two specialist models of the PPS: A protective space, devoted to the defense of the body from external threats, and a working space, where goal‐directed actions take place. They suggested that these two kinds of PPS, while spatially overlapping one another, require distinct sensory and motor processes that follow different principles (de Vignemont & Iannetti).

To conclude, our results show that newborns seem to be able to discriminate between different trajectories taking place in the space immediately surrounding their own body, despite the lack of defensive reactions to impending collision. In particular, our participants showed a visual preference for approaching and colliding stimuli, both when compared with receding and with approaching but non‐colliding stimuli. We speculate that the inconsistency between the visual preference for the colliding trajectory and the lack of defensive behaviors to impending collision at birth could be due to the fact that newborns may lack the experience of dangerous stimuli, necessary to elicit defensive responses (Farroni et al., 2007; Johnson et al., 2015). In fact, newborns might fail to categorize impending collision stimuli as dangerous or generally negative, but, nonetheless, their trajectory might permeate them with a special salience (as suggested by the newborns' visual preferences).

In light of our findings, we suggest that at birth, human infants are already equipped with visual mechanisms capable of processing motion trajectories with respect to the space surrounding their body. Our study demonstrates that the spontaneous preference for stimuli moving directly toward the own body might contribute to the development of the representation of both the bodily self and the PPS, predisposing newborns to perceive their presence in the environment.

## Supporting information


**Movie S1.** First two (out of eight) repetitions of the stimuli presented to the newborns in Experiment 1, Trial A (AC on the left side, R on the right side).

**Movie S2.** First two (out of eight) repetitions of the stimuli presented to the newborns in Experiment 1, Trial B (R on the left side, AC on the right side).

**Movie S3.** First two (out of eight) repetitions of the stimuli presented to the newborns in Experiment 2, Trial A (AC on both the left and the right sides).

**Movie S4.** First two (out of eight) repetitions of the stimuli presented to the newborns in Experiment 2, Trial B (ANC on both the left and the right sides).
